# A physical perspective to understand myelin. I. A physical answer to Peter’s quadrant mystery

**DOI:** 10.3389/fnins.2022.951942

**Published:** 2022-09-26

**Authors:** Yonghong Liu, Wenji Yue, Shoujun Yu, Tian Zhou, Yapeng Zhang, Ran Zhu, Bing Song, Tianruo Guo, Fenglin Liu, Yubin Huang, Tianzhun Wu, Hao Wang

**Affiliations:** ^1^Institute of Biomedical and Health Engineering, Shenzhen Institutes of Advanced Technology (SIAT), Chinese Academy of Sciences (CAS), Shenzhen, China; ^2^Graduate School of Biomedical Engineering, University of New South Wales, Sydney, NSW, Australia; ^3^Key Laboratory of Health Bioinformatics, Chinese Academy of Sciences (CAS), Shenzhen, China

**Keywords:** Peter’s quadrant mystery, oligodendrocyte, E-field, modeling, electrical stimulation

## Abstract

In the development of oligodendrocytes in the central nervous systems, the inner and outer tongue of the myelin sheath tend to be located within the same quadrant, which was named as Peters quadrant mystery. In this study, we conduct *in silico* investigations to explore the possible mechanisms underlying the Peters quadrant mystery. A biophysically detailed model of oligodendrocytes was used to simulate the effect of the actional potential-induced electric field across the myelin sheath. Our simulation suggests that the paranodal channel connecting the inner and outer tongue forms a low impedance route, inducing two high-current zones at the area around the inner and outer tongue. When the inner tongue and outer tongue are located within the same quadrant, the interaction of these two high-current-zones will induce a maximum amplitude and a polarity reverse of the voltage upon the inner tongue, resulting in the same quadrant phenomenon. This model indicates that the growth of myelin follows a simple principle: an external negative or positive E-field can promote or inhibit the growth of the inner tongue, respectively.

## Introduction

Since the pioneering electron microscope (SE) observations of the spiral structure of myelin sheath were conducted between the 1950s and 1980s (BEN GEREN, 1954; Bunge et al., 1989), the ultrastructure and function of the myelin sheath have been paid more attention in neuroscience (Chang et al., 2016; Monje, 2018). The myelin sheath was initially reported as a pure electrical insulator, enabling a “saltatory” impulse propagation (Boullerne, 2016). However, this hypothesis cannot explain many experimental observations in myelin ultrastructures. For example, myelin in the superficial layers of the cortex has diversified longitudinal distribution (Tomassy et al., 2014); myelin sheaths in the peripheral nervous system (PNS) spiral oppositely and the same to its neighbor on the same (Uzman and Nogueira-Graf, 1957; Armati and Mathey, 2013) and adjacent axon, respectively (Richards et al., 1983); and in particular, Peters quadrant mystery (Peters, 1964; Webster, 1971; Fraher, 1972; Waxman and Swadlow, 1976; Berthold and Carlstedt, 1982; Bertram and Schröder, 1993; Schröder, 1996; Traill, 2005) (a mysterious experimental observation showing that the inner and outer tongues of oligodendrocytes tend to locate within the same quadrant) has been observed in many myelin sheaths. These non-trivial ultrastructures imply that the function of the myelin is more than an insulating layer.

An anatomically accurate and biophysically detailed model can improve our understanding of myelin ultrastructures and functions. For example, a coil inductor model of the spiraling structure was used to understand the unique spiraling directions between adjacent myelin sheaths. (Wang et al., 2021) To achieve a positive mutual inductance, the neighboring myelin on the same axon shall have opposite spiraling directions, while the neighboring myelin on the adjacent axons shall have the same spiraling direction. This simulation has been confirmed by SEM (scanning electron microscope) observations (Waxman and Swadlow, 1976).

This study follows the same research paradigm to explore the possible mechanisms underlying the Peters quadrant mystery. In particular, the myelin sheath is modeled as a distributed parameter circuit, and the electric field (E-field) distribution induced by neural electric activities is investigated *in silico*. The simulated E-field was used to explain why the inner tongue and outer tongue of the myelin sheath tend to locate in the same quadrant, a repeatedly observed intriguing phenomenon (Peters, 1964; Webster, 1971; Fraher, 1972; Waxman and Swadlow, 1976; Berthold and Carlstedt, 1982; Bertram and Schröder, 1993; Schröder, 1996; Traill, 2005). The new knowledge gained in this study provides new insights into the relationships between neural electrical activity and myelin growth.

## Peter’s quadrant mystery

**FIGURE 1:**
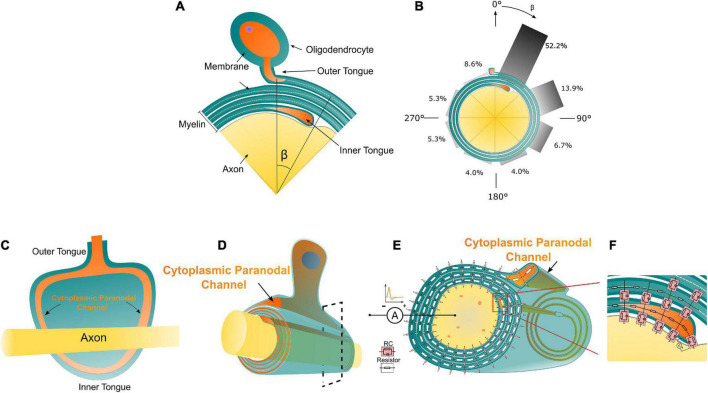
**(A)** The illustration of the Peters quadrant mystery in Oligodendrocytes: there is a relative angle β between the inner and outer tongue. When β is lower than 50°, the inner and outer tongues are considered in the same quadrant. **(B)** The frequency of β within each 45° octant [reproduced from Peter’s observation (Peters, 1964)]. **(C)** The unwrapped myelin sheath with axon showing the connection between the inner/outer tongues and the cytoplasmic paranodal channels. **(D)** An illustrative drawing of an axon myelinated by an oligodendrocyte, the orange part indicates the cytoplasmic paranodal channel connecting the inner and outer tongue; **(D)** The equivalently distributed circuit network model of the cross-section of a myelinated axon. Two kinds of circuit components representing different local electrical properties of the myelin sheath are shown. **(E,F)** The transmembrane capacitance of the growth terminal of the inner tongue.

During axon growth, the myelin wraps around it as a spiral “bandage.” However, there is an interesting tendency for this spiral’s initial and endpoints to occur close together, as if the myelin were insisting on running only complete laps of the arena (Webster, 1971; Traill, 2005). This is analogous to winding rope into a film spool until the rope spills at the angle where the initial “lump” occurs. Initial and endpoints will tend to occur within the same “quadrant” (Figure 1A). Peters first observed this phenomenon in the optic nerves of rodent models in 1964 (Peters, 1964), then further confirmed by multiple studies in visual callosal (Waxman and Swadlow, 1976), dorsal and anterior root axon (Fraher, 1972; Berthold and Carlstedt, 1982), and sural nerves (Bertram and Schröder, 1993; Schröder, 1996). Interestingly, Schwann cell myelination in PNS demonstrated quadrant tendency diminishing gradually with the thickening of myelin (Fraher, 1972). In contrast, myelination of oligodendrocytes in the CNS exhibits a stronger tendency with the thickening of myelin (Peters, 1964).

The actual experimental result of Peter’s quadrant mystery is illustrated in Figures 1A,B with the reproduced data from Peter’s observation (Figure 1B; Peters, 1964). The angle between the outer tongue and the inner tongue is defined as β. As seen in Figure 1B, the occurrence probability of the case when β is within the first quadrant (each quadrant is 45°) is 52.2%, which is much higher than other quadrants. There is an abrupt change of the probability when the inner tongue grows from the last quadrant to the first quadrant.

Peter’s quadrant mystery indicates two points:

1. The outer tongue exerts an effect upon the growth rate of the inner tongue;
2. This effect has an abrupt change when the inner tongue goes through the radial quadrant where the outer tongue is located.

How can the outer tongue affect the growth rate of the inner tongue? This is still an open question that does not seem to have a biological answer (like protein, molecule, and gene) (Traill, 2005). Thus, a physical hypothesis is proposed to build the connection between the growth rate of the inner tongue and the position of the outer tongue. We assume that the electric field (E-field) upon the inner tongue can modulate the inner tongue’s growth rate. A distributed parameter circuit modeling the cross-section of the myelin sheath is built to analyze this E-field.

## Methods

### The circuit simulation

In this study, a cross-section of a myelinated axon is modeled as a distributed parameter circuit, as shown in Figure 1E. The transmembrane parts can be modeled as an RC circuit, while the non-transmembrane parts are modeled as resistors. It is emphasized that the inner tongue and outer tongue are connected with a paranodal channel (Figure 1D), which is a path filled with cytoplasmic liquid and forms a low impedance route. The details of the connection between the inner/outer tongues and the cytoplasmic channels is shown in Figure 1C, showing an unwrapped myelin sheath with the axon. Therefore, in terms of the circuit, the outer tongue and inner tongue are connected with a resistor, whose impedance is low. This low impedance route is critical for the simulation. Since the inner tongue is the growing terminal, the transmembrane voltage of the inner tongue is measured in the simulation, as shown in Figure 1F. The inside terminal of the inner tongue is set as the reference during the measurement.

### The origin of the current source

**FIGURE 2:**
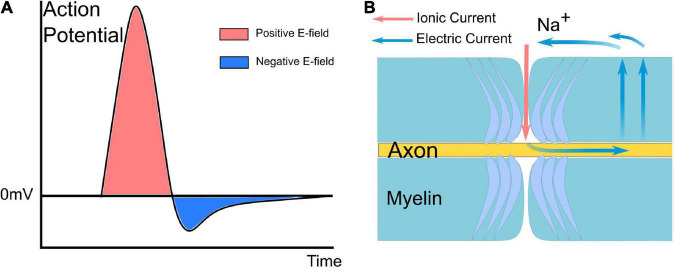
A typical waveform of the action potential and current path between the node of Ranvier and the internode of myelin. **(A)** The action potential is mainly a monophasic positive voltage pulse. **(B)** Based on Kirchhoff’s circuit laws, it can be inferred that there will be an electric current to form a current circle during the inward Na ionic current.

The origin of the current source implemented in the circuit simulation is the action potential. The explanation of the waveform and polarity of the current source is illustrated in Figure 2. Figure 2A shows a typical waveform of the action potential. Since we only consider the absolute voltage change (start from 0 mV rather than −70 mV), the action potential is very similar to a positive monophasic voltage waveform (take the inside terminal of the axon as the reference point in Figure 1E). When an inward ionic current happens at the Ranvier node (the depolarization phase with inward Na^+^ ionic current of the action potential), based on Kirchhoff’s circuit laws, it can be inferred that there will an electric current to form a current circle shown in Figure 2B. Therefore, the E-field across the myelin has a dominant positive component, which is equivalent to a current from the inside to the outside. So the current source applied in Figure 1E has a positive monophasic current waveform with its positive terminal connected with the inside terminal of the axon.

## Results

### The current is more concentrated in the inner-tongue-zone and outer-tongue-zone

**FIGURE 3:**
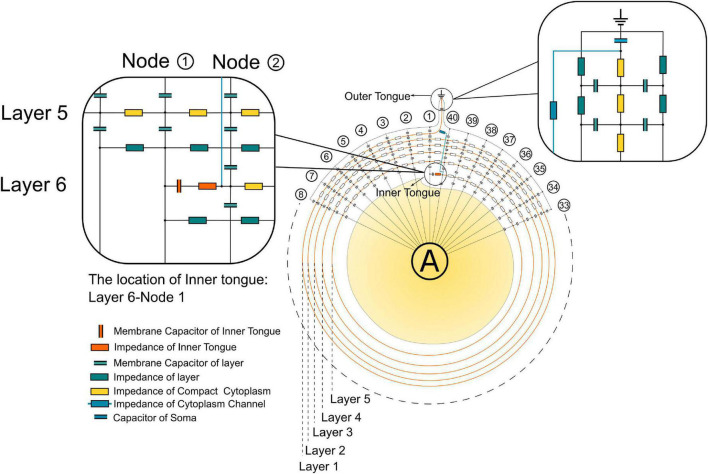
The detailed circuit configuration for modeling.

The detailed circuit configuration of the modeling is shown in Figure 3. The actual parameters of each unit are based on the histological characteristics and measured properties of myelin (Gentet et al., 2000; Liang et al., 2017). The outer tongue is always located at Layer 1. There are 40 nodes for each layer. Then the growth progress of the myelin can be modeled by changing the position of the inner tongue. Thus, for the modeling of dynamic progress of myelin growth, we move the position of the inner tongue. As shown in Figure 3, the inner tongue is located at Layer 6-Node 1, meaning the inner tongue overlaps with the outer tongue at this status, and it is the first unit of Layer 6. At the same time, it was grown from Layer 5-Node 40, the previous state, and will grow to Layer 6-Node 2 in the next state. The voltage change of inner tongue’s membrane capacitor is simulated by all different states of myelin growth progress, from layer 2 to layer 6. All model parameter are described in Table 1.

**TABLE 1:** Electrical parameters of elements used in modeling.

Membrane Capacitor of Inner Tongue	50 nF
Impedance of Inner Tongue	4 Ω
Membrane Capacitor of Layer	1 nF
Impedance of Layer	1 kΩ
Impedance of Compact Cytoplasm	4 kΩ
Impedance of Cytoplasm Channel	10 Ω
Capacitor of Soma	100 nF

**FIGURE 4:**
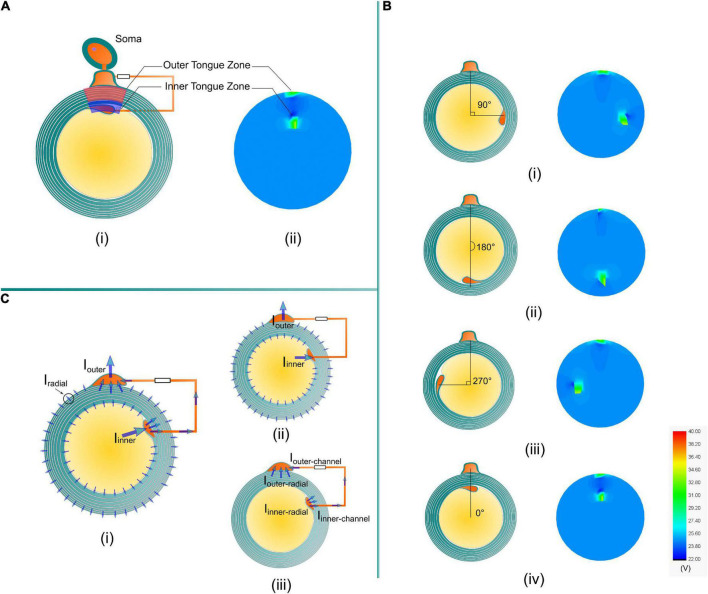
**(A)** The high current zones related to the position of the inner tongue and the outer tongue in the simulation are named the inner tongue zone and outer tongue zone. **(B)** The inner tongue zone moves with the position of the inner tongue when the angles between the inner and outer tongues are **(i)** 90°, **(ii)** 180°, **(iii)** 270°, and **(iv)** 0°. **(C)** A brief illustration of the reason for the two high current zones. **(i)** An illustrative plot of all possible current. **(ii)** The current through the cytoplasmic channel *via* inner and outer tongues is much higher than the radial current in all directions. **(iv)** The current in the cytoplasmic channel will generate a high E-field, making the radial current at the inner and outer tongues more concentrated to form the two high current zones.

The amplitude of the current across each transmembrane capacitor in Figure 1E is recorded and re-distributed into the round shape analogous to the circle of the myelin sheath, as shown in Figure 4A. The current emitted from the axon is not distributed radially identical. Instead, the current is preferentially concentrated at the area close to the inner tongue and outer tongue, called inner-tongue-zone and outer-tongue-zone, respectively (Figure 4A). As shown in Figure 4B, these high current zones move with the position of the inner tongue and outer tongue, showing that these two high current zones are directly induced by the existence of the inner and outer tongues. The cause of these two high current zones is qualitatively explained in Figure 4C.

Since the current source is connected with the inner terminal and outer terminal, it can be considered that the current is emitted from the axon to the outer space of the myelin sheath. The current emission follows a specific distribution, as shown in Figure 4C-i. This distribution can be understood by a two-step analysis shown in Figures 4C-ii,iii. Firstly, due to the existence of the low impedance route, the inner and outer tongue can concentrate more current, shown in Figure 4C-ii:

*I*_*inner*_ ≫ *I*_*radial*_ and *I*_*outer*_ ≫ *I*_*radial*_

The current into the inner tongue is shared between the radial path and the channel path, shown in Figure 4C-iii:


$$I_{i\,n\,n\,e\,r}=I_{i\,n\,n\,e\,r-r\,a\,d\,i\,a\,l}+I_{i\,n\,n\,e\,r-c\,h\,a\,n\,n\,e\,l}$$

It is the same for the current into the outer tongue:


$$I_{o\,u\,t\,e\,r}=I_{o\,u\,t\,e\,r-r\,a\,d\,i\,a\,l}+I_{o\,u\,t\,e\,r-c\,h\,a\,n\,n\,e\,l}$$

Although *I*_*inner–radial*_ is just part of the total current into the inner tongue, due to the current concentration effect, it still can be concluded that:


$$I_{i\,n\,n\,e\,r-r\,a\,d\,i\,a\,l}>I_{r\,a\,d\,i\,a\,l}$$

In general, due to the current concentration effect by the low impedance route connecting the inner and outer tongues, the radial current at the area close to the inner and outer tongue will be higher than that of the other position, resulting in the two high current zones. This is an intuitive and qualitative explanation.

**FIGURE 5:**
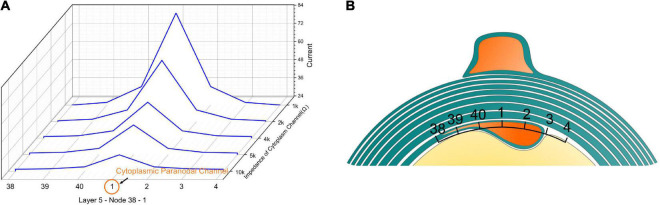
**(A)** The current concentration effect of the cytoplasmic paranodal channel. The resistance represents the cytoplasmic channel connected to Layer 5-Node 1. Then the current on the capacitors of the peripheral region [Layer 5-Node 38, 39, 40, 1, 2, 3, 4 **(B)**] is measured by changing the resistance of the cytoplasmic channel. No matter how high the impedance of the channel is, as long as it exists, the capacitor closer to the channel always has a higher current than the peripheral region.

We also validated that the high current zone is a stable modeling result by changing the impedance of the cytoplasmic paranodal channel, as shown in Figure 5. The resistance represents the cytoplasmic channel is connected to Layer 5-Node 1. Then the current on the capacitors of the peripheral region (Layer 5-Node 38, 39, 40, 1, 2, 3, 4) is measured by changing the resistance of the cytoplasmic channel. As seen, no matter how high the channel impedance is, as long as it exists, the capacitor closer to the channel always has higher current than the peripheral region.

### The radial angle influences the electric voltage on the inner tongue

**FIGURE 6:**
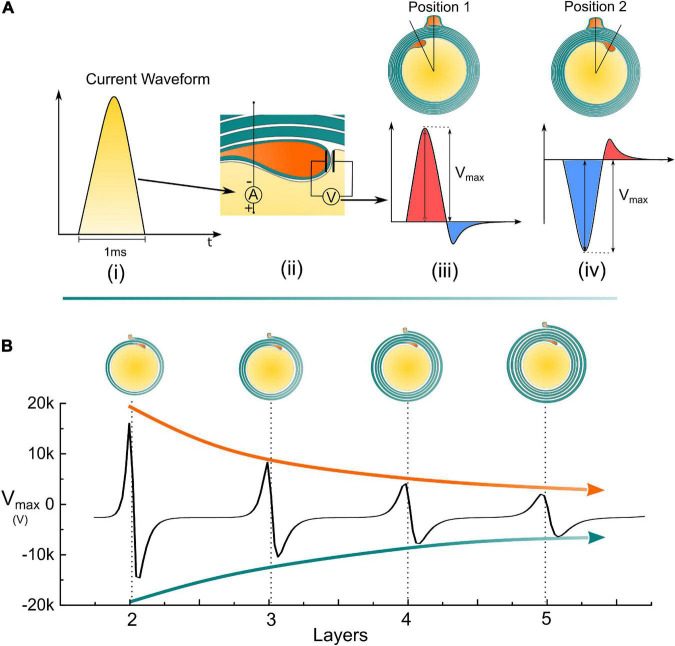
Simulation details and the measured voltage amplitude of the inner tongue. **(A) (i)** A monophasic positive current pulse is used to model the action potential; **(ii)** The detailed configuration of the applied current source and how the voltage on the inner tongue is measured; **(iii)** The illustrative voltage waveform on the inner tongue when the inner tongue is at position 1; **(iv)** The illustrative voltage waveform on the inner tongue when the inner tongue is at position 2; **(B)** The amplitude of the measured voltage amplitude (Vmax) by increasing the number of myelin layers.

Since the inner tongue is the growing terminal, we will focus on how the voltage changes on the inner tongue, which is the voltage measured on the specific capacitor representing the tip membrane of the inner tongue shown in Figure 1F. As explained in Figure 2, the action potential can be approximately considered as a monophasic positive current pulse. The actual applied current waveform in the simulation is also a monophasic current pulse shown in Figure 6A. During the growth of the inner tongue, the voltage amplitude on the inner tongue will have a periodical maximum when the inner-tongue-zone is radially overlapped with the outer-tongue zone (located at the same quadrant in Figure 6B). When the inner tongue is located at position 1 (Figure 6B-i), the voltage waveform as a maximum positive peak. When it is located at position 2 (Figure 6B-ii), the voltage waveform has a maximum negative peak. As seen, the voltage upon the inner tongue has a polarity reverse when it grows from position 1 to position 2. A continuous change of the maximum voltage with the inner tongue growth is shown in Figure 6C, showing a periodic polarity reverse. As seen, the transmembrane voltage of the inner tongue is affected by the relative position between the inner tongue and outer tongue (or the radial angle between the inner and outer tongue). Meanwhile, it has an abrupt change when the inner and outer tongues are in the same quadrant. If the growth rate of the inner tongue is modulated by the polarity and amplitude of this voltage, the same quadrant mystery can have a simple answer, as explained below.

### The voltage polarity reverse

**FIGURE 7:**
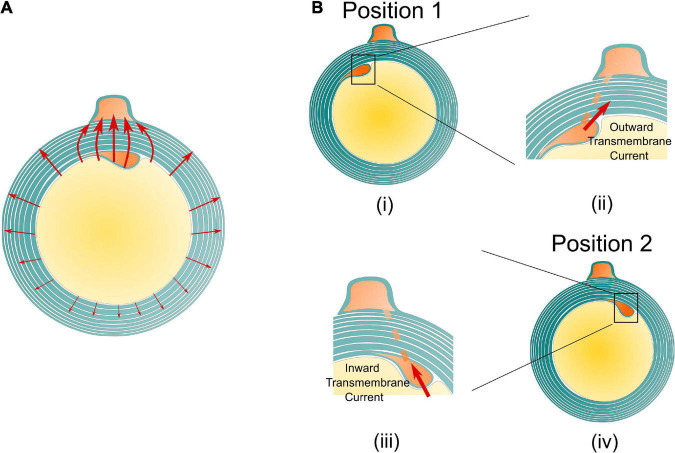
The illustrative current flow on the cross section of the myelin sheath. **(A)** The current from the inner tends to flow toward the outer tongue. **(B)** An illustrative drawing explains the reason for the polarity reverse. When the inner tongue is at position 1 **(i)**, the current from the inner tongue to the outer tongue forms an outward transmembrane current on the inner tongue **(ii)**; when the inner tongue is at position 2 **(iii)**, the current from the inner tongue to the outer tongue forms an inward transmembrane current on the inner tongue **(iv)**.

An illustrative drawing to explain the polarity reverse is shown in Figure 7. When the inner tongue and the outer tongue are located in the same quadrant, the two high-current zones will have interaction, forming a directional current flow from the inner tongue toward the outer tongue (Figure 7A). When the inner tongue is located at position 1, the radial current of the inner tongue, *I*_*inner–radial*_, toward the position of the outer tongue will form a transmembrane current upon the inner tongue with an outward direction shown in Figure 7B-i, which is equivalent to an externally applied negative E-field. When the inner tongue is located at position 2, this transmembrane current has an inward direction, which is opposite to the situation of position 1 (Figure 7B-ii) and is equivalent to an externally applied positive E-field. This is the reason for the polarity reversed in Figure 6.

### The explanation to Peter quadrant mystery

**FIGURE 8:**
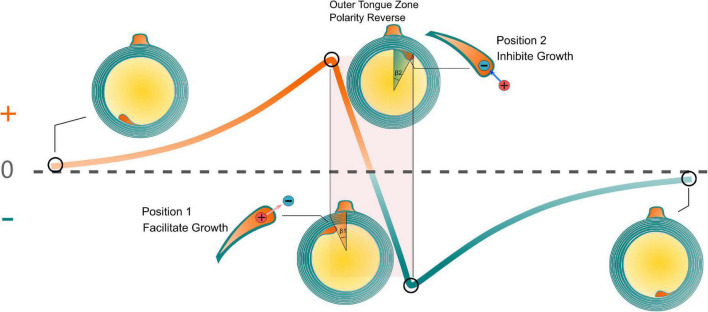
The relationship between the transmembrane voltage of the inner tongue and its radial position. The growth rate is correlated with the polarity (direction) and amplitude of the transmembrane E-field.

The relationship between the transmembrane voltage of the inner tongue and its radial position is illustrated in Figure 8. It is emphasized that the curve in Figure 8 is an illustrative drawing, not an accurate duplication from the simulation results. When the inner tongue locates in position 1 (entering the outer-tongue-zone), the transmembrane E-field of the inner tongue reaches the maximum outward value. In Peter’s observations, position 1 showed the lowest occurrence frequency (Peters, 1964), indicating the fastest growth rate. With further growth, the inner tongue will reach position 2 (leaving the outer-tongue-zone). The transmembrane E-field of the inner tongue reaches the maximum inward value. The occurrence frequency at this position is the highest, indicating the slowest growth rate. Therefore, we can conclude that the growth rate is correlated with the polarity (direction) and amplitude of the transmembrane E-field. An outward E-field can facilitate growth, while an inward E-field can inhibit growth. In other words, an extracellular negative E-field can promote myelin growth, while an extracellular positive E-field can inhibit myelin growth.

**FIGURE 9:**
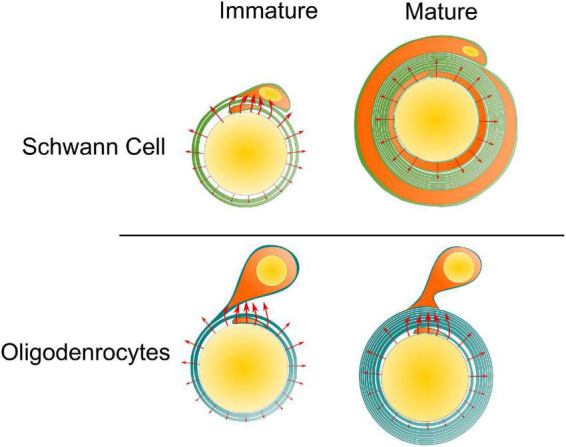
The same quadrant phenomenon for Schwann cell and Oligodendrocyte. For the Schwann cell, at its mature state, its inner tongue and outer tongue form two circles, eliminating the occurrence of the two high-current zones. So the same quadrant phenomenon tends to disappear with growth.

Our simulations suggested that the existence of the paranodal channel connecting the inner and outer tongue forms two high-current zones. When these two high-current-zones are getting close, which happens when the inner and outer tongues are located within the same quadrant, the voltage upon the inner tongue will have a maximum amplitude and a polarity reverse, resulting in a minimum growth rate at position 2. So the inner tongue tends to stay at position 2, observed as the same quadrant phenomenon. Interestingly, this phenomenon does not only appear in Oligodendrocytes (Peters, 1964; Waxman and Swadlow, 1976) but also exists in the early stage of the myelination by Schwann cells in PNS (Fraher, 1972). A comparison of the difference between Oligodendrocytes and Schwann cells is shown in Figure 9 to explain this experimental observation. For oligodendrocytes, the inner tongue and outer tongue are connected with paranodal channels. For Schwann cells, apart from the paranodal channels, there are also other cytoplasmic channels, called Schmidt–Lanterman incisures (SLI), located along the whole internode span. All these cytoplasmic channels connect the inner and outer tongues of the Schwann cells, providing low impedance routes. Thus, the same quadrant phenomenon also happens during the growth of Schwann cells. However, it is known that for a mature Schwann cell, its inner tongue and outer tongue form two radial circles shown in Figure 9, rather than just occupying a certain radial angle, which is the situation of Oligodendrocytes. Therefore, the same quadrant tendency will diminish for a mature Schwann cell (Gomez-Sanchez et al., 2017).

### A possible explanation for g-ratio

The g-ratio is the ratio of the inner axonal diameter to the total outer diameter, including the myelin sheath (Chomiak and Hu, 2009). The g-ratio ranged from 0.72 to 0.81 in CNS, and 0.46 to 0.8 in PNS. However, if the axon diameter is less than 0.4 μm, it will fail to form the myelin sheath (Waxman and Bennett, 1972), indicating the key role of the axonal physical properties in terminating the growth of myelin. Although there are still controversies (Smith and Koles, 1970; Waxman and Bennett, 1972), earlier studies suggested the contribution of g-ratio in modulating conduction velocity (Sukhorukov et al., 1993). However, this theory fails to build the connection between the signal propagation and the inner tongue, which is the growth terminal of the myelin. In this study, the modeled E-field tends to decrease by increasing the layers of myelin layers. Thus, when the E-field is lower than a certain threshold, the growth of the inner tongue will be automatically terminated. Thus, this theory indicates the potential correlation between the g-ratio and the E-field on the inner tongue. In our next study, a more detailed model is proposed to explain the g-ratio phenomenon (Liu et al., 2021).

## Discussion

### The same quadrant phenomenon does not happen at the early stage of myelination

There is another evidence to support our theory that the same quadrant phenomenon is deeply linked with cytoplasmic channels. During the myelination, the myelin will have a compaction process to form these cytoplasmic channels connecting the inner and outer tongues. Therefore, the same quadrant phenomenon shall not happen at the early stage of myelination since the myelin is not compact yet. This agrees with the experimental observation (Peters, 1964). The same quadrant phenomenon is not observed for optical nerves of 7 days postnatal rats when the number of myelin lamellae is not higher than 3. While for optical nerves of postnatal rats older than 14 days, when the number of myelin lamellae is higher than 4, the same quadrant phenomenon can be clearly observed.

### The same quadrant phenomenon also does not only happen in the section of the same quadrant

As depicted in Figure 1B, the occurrence of the inner tongue’s position is the maximum at about 1 o’clock direction and gradually decreases in the clockwise direction. From the analysis in Figure 6, the voltage upon the inner tongue also reaches the negative maximum at about 1 o’clock direction. Then, it gradually increases to the positive maximum in the clockwise direction. So our model can explain the same quadrant phenomenon and the clockwise changing trend, duplicating all minor details of the experimental observation.

### Comparison with previous studies on explaining the same quadrant phenomenon

The same quadrant phenomenon has long been considered a mystery (Chang et al., 2016). Although it has been observed and validated by many studies (Peters, 1964; Webster, 1971; Fraher, 1972; Waxman and Swadlow, 1976; Berthold and Carlstedt, 1982; Bertram and Schröder, 1993; Schröder, 1996; Traill, 2005), only two previous works tried proposing possible mechanisms (Traill, 2011; Fabbri, 2015). One is proposed by I. M. Fabbri (2015). In his work, the myelinated nerve is modeled as a spiral coaxial cable to transmit the neural signal as an electromagnetic wave. Suppose the inner and outer tongues are located very close to each other. In that case, the myelin sheath will be more like a spiral geometry, which is preferred for better handling power throughout the nervous system. Another is proposed by Robert R. Traill (2011), who considers the myelinated nerve an optical fiber to transmit IR radiation. If the number of myelin lamellae is integral, the myelin sheath can form a perfect circle, which is beneficial for IR radiation as a standing wave to propagate. But in Robert R. Traill’s model, the outer tongue is the growing terminal, which is biologically incorrect. Both of these two models fail in several aspects.

1. The same quadrant phenomenon does not mean myelin lamellae should be an integral number. The integral number means the inner tongue should stop its growth at position 1 in Figure 6, which these two models presume. However, it is clearly mentioned in the original experimental observation that the inner tongue stops at position 2, which is a bit over the outer tongue. Our model explains that the inner tongue should stop at position 2 due to the minimum growth rate.
2. As mentioned in section “The same quadrant phenomenon does not happen at the early stage of myelination”, in our model, the same quadrant phenomenon does not happen in the early stage of myelination when the myelin sheath is not compact, which agrees with the experimental observation. However, both of these models do not account for it.
3. The gradual change along the clockwise direction, as explained in section “The same quadrant phenomenon also does not only happen in the section of the same quadrant,” is not accounted for in these two models.
4. Both models try to explain the phenomenon in terms of function rather than reason. An observed phenomenon always has a reason to happen but does not necessarily serve a specific function. Explaining a phenomenon in terms of function is not so reliable since the assumed function may not really exist. Our model gives the origin of the same quadrant phenomenon, explaining how this same quadrant phenomenon is formed.

### The physical perspective to study the myelin development

The conventional research paradigm from biological and chemical perspectives is quite limited for neuroscience, especially for the myelin study. Now we know quite a lot of ultrastructures of myelin sheaths, such as the non-random spiraling directions between neighboring myelin sheaths (Uzman and Nogueira-Graf, 1957; Richards et al., 1983; Bunge et al., 1989; Armati and Mathey, 2013), the same quadrant phenomenon (Peters, 1964), g-ratio (Stikov et al., 2015) and radial sorting (Pereira et al., 2009). All these ultrastructures were repeatedly observed and validated by many studies and yet have reasonable explanations. A possible reason is that these ultrastructures imply an effect of action at a distance, whose explanations are beyond biology and chemistry. The effect of action at a distance is a term in physics, meaning that one object can exert an effect upon another object without physical contact. Normally, this effect of action at a distance is realized by a field. Let’s take the same quadrant phenomenon in this study as an example. There are so many compact myelin lamellae between the inner and outer tongues. But the position of the outer tongue determines where the inner tongue should stop its growth without physical contact. It is difficult to imagine any protein or receptor can induce this result. But the electric field can easily build the logical connection between the positions of inner and outer tongues. Another successful case is the explanation of the non-random spiraling directions between neighboring myelin sheaths by the electromagnetic field (Wang et al., 2021). It is known that on the same axon, if one myelin sheath spiraling direction, from inner tongue to outer tongue, is clockwise, then the neighboring myelin sheath will have the opposite spiraling direction, which is anti-clockwise (Uzman and Nogueira-Graf, 1957). Again, it is almost impossible to imagine a protein or a receptor on one myelin sheath that can sense the spiraling direction of the neighboring myelin sheath and then determine the spiraling direction of itself. In our previous model, the spiraling of the cytoplasmic channel in the myelin sheath is considered a coil inductor to generate a magnetic field from the current of action potential activation. Due to the mutual inductance between the cytoplasmic channels in adjacent myelin sheaths as coil inductors, the current in one myelin sheath will generate an induced current in the neighboring myelin sheath by electromagnetic induction. Since a positive mutual inductance is beneficial for neural signal propagation, the spiraling directions between adjacent myelin sheaths should always be opposite, as observed in the experiments. But this explanation in terms of function is incomplete since it does not give the reason for the formation. However, by knowing the effect of electric field modulation on the myelin growth proposed in this study, it can be inferred that the electric field also induces this non-random spiraling. The current induced by the adjacent myelin sheath can affect the myelin growth when the first lamella is formed. One terminal grows faster due to the induced current and then becomes the inner tongue, thus determining the spiraling direction. In our next study, we will further show how this model is further extended to account for other ultrastructures such as g-ratio and radial sorting.

### The relationship between neural activity and myelin development

It is known that myelin forms the white matter, a major portion of our brain. It plays a critical role in neural signal propagation and memory (Fields and Bukalo, 2020). Meanwhile, most degenerative neural diseases are accompanied by demyelination or myelin degeneration. Therefore, investigating the development mechanism and regeneration method of myelin is an important topic in neuroscience and treating degenerative neural diseases. Currently, one of the most promising directions to decode the development mechanism of myelin is neural activity-dependent myelination (Fields, 2015). Many studies show that both myelination and demyelination are deeply linked with action potentials. But the detailed interaction mechanism between the action potential and the myelin growth is yet to be elucidated. However, this study proposes a definite principle of E-field modulated myelin growth, showing that the electric field can exert both promoting and inhibitory effects. Considering that neural activity is mainly a changing E-field, our theory provides the clue to unveil the secret of myelin development. Moreover, our model may also explain the direct cause of some degenerative neural diseases, such as Parkinson’s disease, as abnormal neural activities. We will make a more detailed discussion about this part in our next work (Liu et al., 2021).

### The limitation of our model

The major limitation of our model is that, in the current stage, the model is qualitative rather than quantitative. The qualitative modeling result is determined by the circuit structure, which is a circular capacitor network with a low impedance route connecting the positions of inner and outer tongues, as shown in Figure 3. As long as this circuit structure is the same, the qualitative modeling result shown in Figure 6B and Figure 8 is not affected by the actual circuit parameters. Although our model can derive the principle of electrical modulation on myelin growth, the actual amplitude of the voltage/E-field required to affect the myelin growth remains unknown. But it is also quite difficult to further improve the model due to several factors.

1. The first one is the parameter rescale issue. A myelin sheath is a 3D structure with a certain longitudinal length, while our circuit model is 2D. How to rescale the parameter of an electrical component representing a 3D object is always a question. Therefore, it is difficult to precisely determine the actual value of each component in the circuit.
2. The active properties of the myelin sheath are neglected. Our model only considered the passive properties of the myelin structure (that is, the RC properties) without including active ionic mechanisms such as potassium ion channels (Wilson and Chiu, 1990) and radial components (Peters, 1961). However, the contribution of active conductance in the voltage generation upon the inner tongue is still an open question without experimental details of potassium channel density distributed in myelin.

## Conclusion

The physical origin of the same quadrant mystery is the preferential E-field distribution on the cross-section of the myelin. Since actional potentials induce E-field, it explains the relation between neural electric activity and the ultrastructure of myelin. Furthermore, the preferential E-field distribution resulting from the breaking of the central symmetry by the outer tongue explains the difference of the “same quadrant” observation between Oligodendrocytes in CNS and Schwann cells in PNS. Meanwhile, this study also reveals the physical factor that modulates myelin growth: an extracellular negative or positive E-field can promote or inhibit myelin growth, respectively. Finally, the computational approach can probe neuronal ultrastructures at a resolution far beyond the current state-of-the-art biological experiments, providing a promising tool to explore neuroscience from a physical perspective.

## Data availability statement

The original contributions presented in this study are included in the article, further inquiries can be directed to the corresponding author/s.

## Author contributions

HW proposed the theory. YL carried out the modeling process. TG helped refine the theory and improve the writing. WY, TZ, YZ, RZ, BS, FL, YH, and TW contributed to the reference collection, idea discussion, and early state of the theory establishment. SY helped plot figures and search for the references for manuscript revision. All authors contributed to the article and approved the submitted version.
